# The Prognostic Value of SOX2 Expression in Non-Small Cell Lung Cancer: A Meta-Analysis

**DOI:** 10.1371/journal.pone.0071140

**Published:** 2013-08-19

**Authors:** Yansu Chen, Yefei Huang, Yulin Huang, Junjie Chen, Shouyu Wang, Jianwei Zhou

**Affiliations:** Department of Molecular Cell Biology and Toxicology, Jiangsu Key Lab of Cancer Biomarkers, Prevention & Treatment, Cancer Center; School of Public Health, Nanjing Medical University, Nanjing, Jiangsu Province, People's Republic of China; MOE Key Laboratory of Environment and Health, School of Public Health, Tongji Medical College, Huazhong University of Science and Technology, China

## Abstract

**Objective:**

To investigate the association of SOX2 expression in tumor with clinicopathological features and survival of non-small-cell lung carcinoma (NSCLC) patients.

**Methods:**

Publications assessing the clinicopathological characteristics and prognostic significance of SOX2 in NSCLC were identified up to May 2013. A meta-analysis of eligible studies was performed using standard statistical methods to clarify the association between SOX2 expression and these clinical parameters.

**Results:**

A total of eight studies met the inclusion criteria. Analysis of these data showed that SOX2 expression was positively associated with squamous histology, (pooled OR = 5.26, 95% CI: 1.08–25.6, *P* = 0.040). Simultaneously, we also found that SOX2 expression was positively associated with overall survival (pooled HR = 0.65, 95% CI: 0.47–0.89, *P* = 0.007, random-effect).

**Conclusions:**

SOX2 expression in tumor is a candidate positive prognostic biomarker for NSCLC patients.

## Introduction

Lung cancer (LC) is the most commonly diagnosed cancer as well as the leading cause of cancer death worldwide [1]. The main types of LC are small-cell lung carcinoma (SCLC) and non-small-cell lung carcinoma (NSCLC). Among all LC cases, NSCLC accounts for approximately 85% [2]. Though significant diagnostic and therapeutic improvements have been made for NSCLC, the prognosis is still suboptimal, with an overall five-year survival rate of less than 15% [3]. Recent advances have provided provocative insights in the biology of NSCLC that may result in the discovery of biological markers, that are urgently needed for guidance on postoperative surveillance and therapeutic decisions [4].

SRY (sex determining region Y)-box 2, also known as SOX2, is one of the key transcriptional factors that control the unique properties of stem cells self-renewal and pluripotency [5], [6] and play a critical role inmaintaining the stem cell-like phenotype in cancer cells [7]–[9]. Over-expression of SOX2 in NSCLC cells stimulates cellular migration and anchorage-independent growth while SOX2 knockdown impairs cell growth [10]–[12]. Recently, a number of studies have reported the contribution of SOX2 to tumorigenesis and itscorrelation with clinical progression of various types of tumors, including human breast cancer [13], rectal cancer [14], prostate cancer [15] and NSCLC [16], [17]. SOX2 gene amplification is frequently up-regulated in NSCLC [18], [19] and is associated with poor prognosis [17], but recently results show that high SOX2 levels predict better outcome in NSCLC [16]. These conflicting results on the detection, clinical pathologic features and progression of SOX2 positive expression could indicate limited availability of samples resulting in variations in the clinical significance of the results and the need for overall analysis. Considering the putative role of SOX2 in the prognosis and prediction of outcome in NSCLC, a meta-analysis was conducted to determine the association between SOX2 and common clinical and pathologic features of NSCLC.

## Materials and Methods

### Publication search

The electronic database of PubMed was searched for studies that investigated the association of clinicopathological parameters and prognosis with SOX2 expressionin NSCLC to be included in the present meta-analysis upto May 14, 2013. Search terms were “lung cancer” and “SOX2”. The published studies that were included in this meta-analysis should meet the following criteria: (1) the histologic type of the tumors was NSCLC; (2) they assessed the relationship between SOX2 expression and clinicopathological features and/or survival; and only full peer reviewed papers have been published as full texts. There were no limitations on language nor on patient numbers. When multiple NSCLC cohorts were used to validate the same results in one paper, groups using the same detection methods were merged as one group. Publications re-using datasets from the same population, the article with more extracted details was included. Studies that did not meet all inclusion criteria were excluded.

### Data extraction

Two reviewers checked all potentially relevant studies independently to minimize bias and to improve the reliability. The following characteristics were extracted from eligible studies: name of first author, name of journal, year of publication, sample size, test method, cut-off value, age, gender, smoking status, histologic type, differentiation, lymph node metastasis, stage as well as the expression-related survival. In case the prognosis was only plotted as Kaplan-Meier curve, the software GetData Graph Digitizer 2.24 (http://getdata-graph-digitizer.com/) and HR digitizer software Engauge 4.0 were applied to digitize and extract the data. Briefly, save the Kaplan-Meier curve as a graph andopen the graph in the software GetData Graph Digitizer 2.24 and Engauge Digitizer 4.0, then set the scale (coordinate system) and finally digitize the points of Kaplan-Meier curve manually.

### Statistical analysis

All the statistical analyses were performed using Stata/SE 10.0 for Windows (Stata Corporation, College Station, TX, USA). Pooled estimates of odds ratios (OR) with their 95% confidence intervals (CI) were used to estimate the association between SOX2 expression and the clinical parameters of NSCLC, including age, gender, smoking status, histologic type, differentiation, lymph node metastasisas well as stage. Pooled estimates of hazard ratios (HR) with their 95% CI were used to estimate the association between SOX2 expression and survival outcome of NSCLC. The statistical heterogeneity within studies was tested with the chi-squared based Q-test (*P*>0.10) and I^2^ (I^2^<50%, no heterogeneity), fixed-effects model was used. Otherwise, the random-effects model was used. To explore the possible heterogeneity among different studies, the important variables of population, histologic type and sample size were examined in a meta-regression model. The between-study variance (τ^2^) was used to quantify the degree of heterogeneity among studies, and the percentage of τ^2^ was used to show the extent of the explained heterogeneity of the characteristics [20]. Evidence of publication bias was analysed by Egger's and Begg's test, the potential publication bias was deemed significant with *P*<0.05.

## Results

### Description of studies

**Figure 1:**
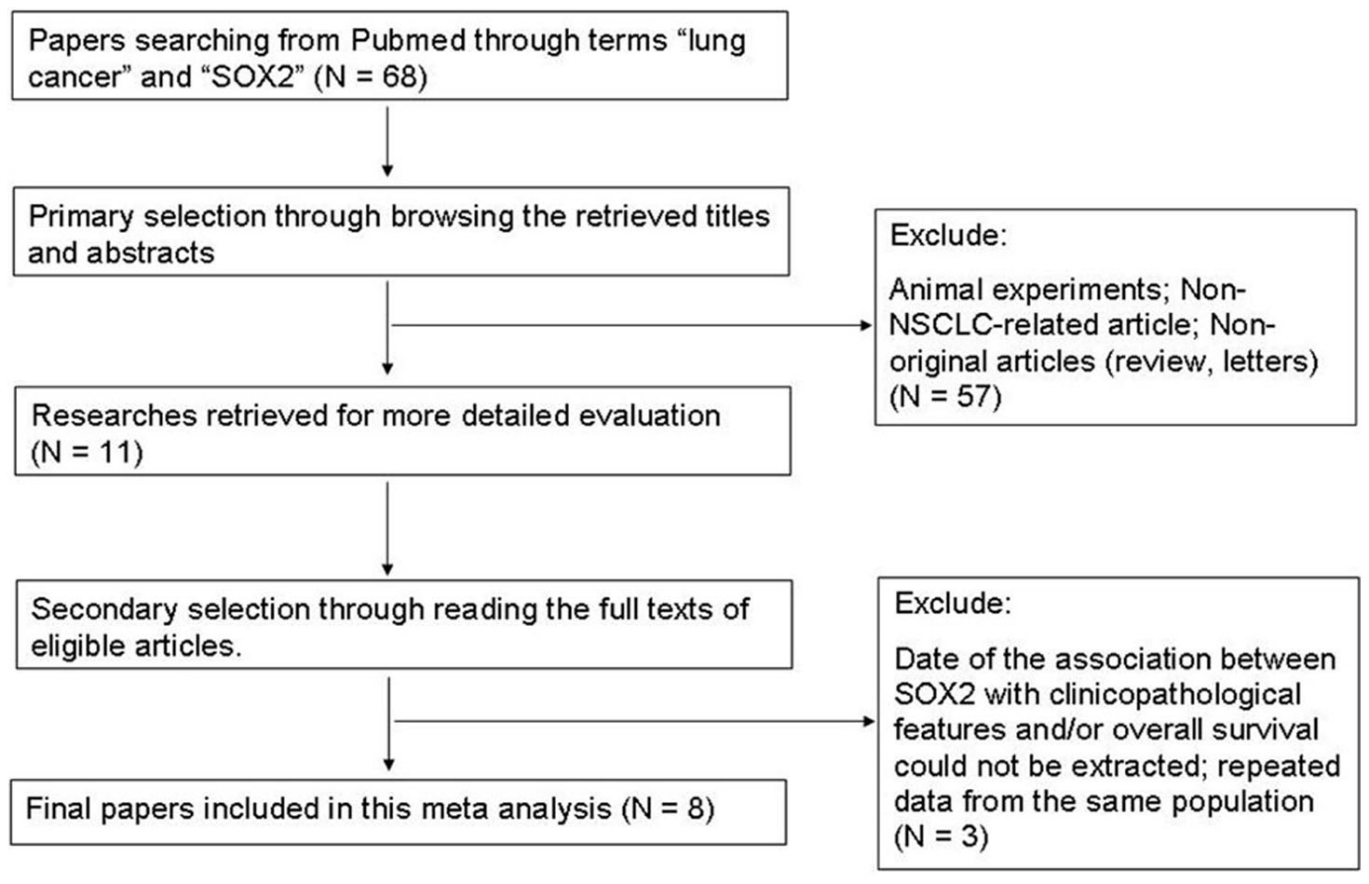
Flow chart for selection of studies for inclusion in this meta-analysis.

Combined search in PubMed on the terms “lung cancer” and “SOX2” retrieved 68 hits, and when excluding animal experiments, non-NSCLC-related studies, non-original articles, or lack of data on the association of SOX2 with clinicopathological features and/or overall survival and repeated data from the same population [21], [22], only 8 publications met the inclusion criteria for the present analysis (Figure 1). Two of these studies lacked information on survival and follow up and thus could not be used for survival analysis. The sample sizes ranged from 44 to 758 patients. Expression of SOX2 was evaluated by immunohistochemistry (IHC) in four studies, quantitative real-time polymerase chain reaction (qPCR) in two studies, fluorescence in situ hybridization (FISH) assay and tissue immunofluorescence (IF) in one paper, respectively. The detailed outline of the parameters of the included studies is shown in Table 1.

**Table 1:** Main characteristics and results of the eligible studies.

No. of paper	First author	Journal	Year	Country	Methods	Duration of Follow-up (months)	Cut-off point (high/low)	No.of patients	No.of Deceased/Alive
(1)	L. M. Sholl	Am J Surg Pathol	2010	USA	IHC	136	>5% (52/52)	104	57/47
(2)	P. Yuan	PLoS One	2010	USA	IHC	-	SCC (high >270, low <140) (29/11); ADC (high>193, low <10) (9/8)	57	-
(3)	Y. Lu	PLoS One	2010	USA	IHC	48	>5% (19/89)	89	33/13*
(4)	T. Wilbertz1	Mod Pathol	2011	Switzerland/USA	FISH	169	>30% (225/533)	758	235/33*
(5)	Y. R. Cai	Oncol Lett	2011	China	qPCR	-	Ratio>M +2 SD (30/85)	115	-
(6)	X. X. Li	Int J Mol Sci	2012	China	IHC	12	≥10% (31/13)	44	31/13
(7)	H. Sasaki	Exp Ther Med	2012	Japan	qPCR	120	Ratio>4 copies(42/85)	127	91/36
(8)	V. Velcheti	PLoS One	2013	Greece/USA	IF	60	Score>193(418/229)	647	392/255

**Abbreviations**: IHC, immunohistochemistry; qPCR, quantitative real-time polymerase chain reaction; FISH, fluorescence in situ hybridization; IF, immunofluorescence; SCC, squamous cell carcinomas; ADC, adenocarcinomas; M, mean; SD, standard deviation.

**Note**:

* only with SCC patients;

### Correlation of SOX2 expression with clinicopathological characteristics

The main results of the meta-analysis are summarized in Table 2. In some studies presented data on clinicopathological features could not be extracted and only parameters were present in >3 papers, the meta-analysis was performed. There was no correlation between SOX2 expression and clinicopathological parameters such as age, sex, smoking, lymph node metastasis andstage. However, SOX2 expression was positively correlated with squamous cell carcinomas (SCC) compared with adenocarcinomas (ADC) (pooled OR = 5.26, 95%CI:1.08–25.6, *P* = 0.040).

**Table 2:** Main results for meta-analysis between SOX2 and clinicopathological Parameters.

clinical parameters	No. of studies	overall OR (95%CI)	Heterogeneity test (Q, I^2^, *P*)
Sex (male vs. female)	(1),(4),(5),(6),(7)	1.49 (0.69–3.23)	10.6, 62.3%, 0.312 (random-effect)
Age (>60 vs. < = 60)	(1),(5),(6),(7)	1.30 (0.84–2.01)	3.88, 22.7%, 0.236 (fixed-effect)
Smoking status (yes vs. no)	(1),(4),(5),(7)	2.40 (0.95–6.05)	6.79, 55.8%, 0.065 (random-effect)
Histology (SCCvs. ADC)	(2),(3),(4),(5),(6),(7)	5.26 (1.08–25.6)	78.2, 93.6%, 0.040 (random-effect)
Differentiation (poor vs. well)	(5),(6),(7)	1.97 (0.96–4.05)	0.49, 0.0%, 0.066 (fixed-effect)
Lyphmnodemetastsis (N1 vs. N0)	(4),(5),(7)	1.30 (0.85–1.97)	1.48, 0.0%, 0.226 (fixed-effect)
Stage (III/IV vs. I/II)	(4),(6),(7)	0.89 (0.55–1.44)	2.08, 3.9%, 0.621 (fixed-effect)

**Abbreviations**: SCC, squamous cell carcinomas; ADC, adenocarcinomas; OR, odds ratios.

### Impact of SOX2 expression on overall survival of NSCLC

**Figure 2:**
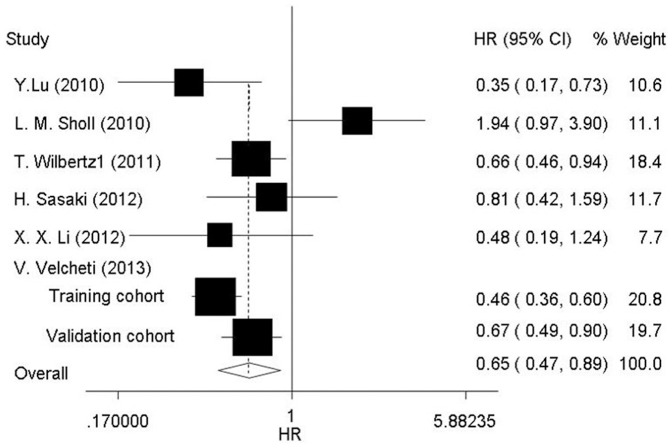
Forest plot showed that the SOX2 expression was associated with overall survival of NSCLC.

The different results obtained from previous studies on the impact of SOX2 expression on overall survival. Since in some papers, the association of SOX2 expression with overall survival of NSCLC patients was calculated in different subgroups, such as those divided by gender in L. M. Sholl2010 [17], and SOX2 expression divided by no, low and high amplification in T. Wilbertz12011 [23], the group with more population was selected for HR evaluation. Additionally, both of two independent studies in V. Velcheti (2013) [16] were selected for further pooled HR analysis, due to the samples recruited from different countries. The accumulative overall survival rates of SOX2-positive and SOX2-negative NSCLC patients were 36% (282/789) and 26% (115/447), respectively. The pooled HR of the overall survival was 0.65 (95% CI: 0.47–0.89, *P* = 0.007, random-effect, Figure 2), with an I^2^ of 68.4%.

### Test for heterogeneity

There was significant heterogeneity for histology assessment (I^2^ = 93.6%) and survival evaluation (I^2^ = 68.4%). Then, we assessed the source of heterogeneity for additive model by population (Asian vs. the others), histological type (SCC vs. ADC vs. both SCC and ADC) and sample size (>100 vs. <100). For the histology, we observed that population (*χ* ^2^ = 44.96, df = 1, *P*<0.001) and sample size (26.56, 1, 0.001) contributed to substantial heterogeneity. For the survival, histological type (12.36, 2, 0.002) but not population (0.25, 1, 0.619) or sample size (1.22, 1, 0.268) were found to contribute to substantial heterogeneity. Moreover, the estimated between-study variance (τ^2^) was used to quantify the degree of heterogeneity among studies by meta-regression analysis. It was shown that population and sample size could explain 75.5% of the τ^2^ for the histology assessment, and histological typecould explain 80.2% of the τ^2^ for the survival assessment.

### Sensitivity analyses

We also performed sensitivity analyses to evaluate the stability of the results. Our results showed that the heterogeneity for histology was effectively decreased by exclusion of the study of T. Wilbertz12011 [23] (I^2^ = 69.3%), but the pooled OR was not significantly influenced. For the survival, we moved an independent study by L. M. Sholl2010 [17], the heterogeneity was effectively decreased (I^2^ = 31.6%), but the pooled HRwas not effectively influenced, which suggested that the results of this meta-analysis are stable.

### Publication bias

**Figure 3:**
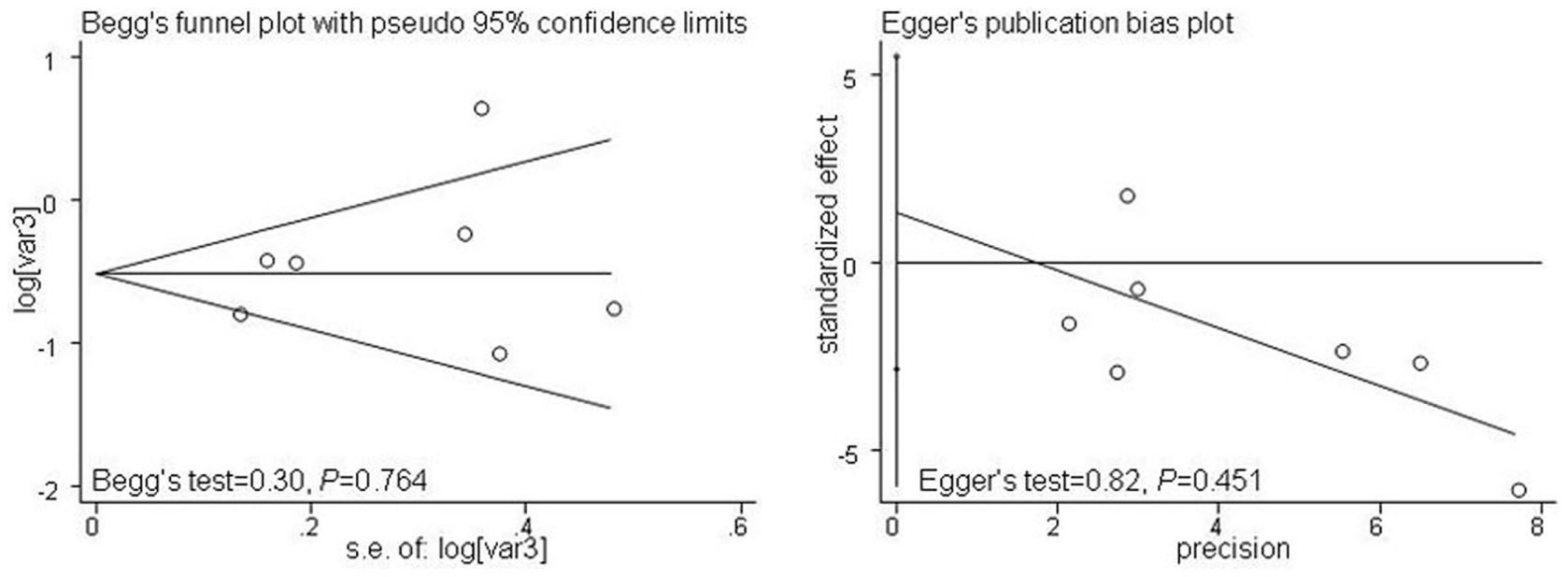
Begg's and Egger's funnel plot estimated the publication bias of the included literature.

Begg's funnel plot with pseudo 95% confidence limits and Egger's test were performed to estimate the publication bias of the included literature (Figure 3). Begg's and Egger's test did not reveal any evidence of obvious asymmetry in the overall meta-analysis of all studies.

## Discussion

NSCLC is the leading cause of cancer death, with an overall five-year survival rate of less than 15% [1], [3]. New biological markers of NSCLC carcinogenesis may provide important progress in clinical decision making [4]. Emerging evidences have suggested functional molecules involved in cell-cycle control, DNA repair, proliferation, apoptosis that may modulate response to platinum-based chemotherapy and serve as promising biomarkers for individualized chemotherapy and prognosis of NSCLC patients [24]–[27].

SOX2 expression plays a critical role incell cycle control, DNA damage response and long-term self-renewal in neural stem cells [28], [29]. Moreover, several studies and our data have identified that SOX2 expression correlated with tumorigenesis, chemoresistance, and maintaining the stem cell-like phenotype in cancer cells [7]–[9], [30]. Recently, it has been reported that SOX2 expression may serve as a promising biomarker in prognosis of NSCLC [7], [16]–[18], [21], [23], [31], [32], however, these results were contradictory. Therefore, the present meta-analysis, is a quantitative approach to statistically integrate and analyze the association between SOX2 expression and NSCLC clinicopathological characteristics and overall survival.

Recent results show that SOX2 is more frequently up-regulatedin SCC than ADC patients [7], [16], [23], which was accordant with our meta-analysis that SOX2 expression was positively associated with SCC compared with ADC. Though SOX2 over-expression was reported to associate with lower tumor grade, smaller tumor size and lower probability of invasion and metastasis [23] and former and current smoking status [7], here our results showed that SOX2 positive expression was not correlated with these clinicopathological parameters. Simultaneously, reports also indicate that SOX2 amplification and over-expression have significant, non-significant and contradictory association with outcome in NSCLC [16], [23], [31]. For example, SOX2 over-expression was recently reported to beassociated with better outcome in SCC [23], but with poor outcome in early stage lung ADC [17]. Our results found that despite of histology, high SOX2 expression was a positive prognostic biomarker. This finding was supported by the notion of Velcheti et al. that the association SOX2 expression with better survival is independent from the histological subtype [16].

This systematic review has some limitations. First, the number of included studies, as well as the included NSCLC patients in each study, is relatively small. Secondly, though no significant heterogeneity across studies was detected in our study, we could not fully neglect potential heterogeneity. The subgroup and meta-regression analysis were used to assess the sources of heterogeneity. When these studies moved, the heterogeneity was decreased but the pooled results were not influenced, which suggested these data are stable. Thirdly, the methods used for the assessment of the level of SOX2 expression in NSCLC patients differed among these studies. Besides, there were some differences among these studies in cutoff values of defining the specimens as positive SOX2 expression or amplification. The new studies with same cutoff values must be recruited and combined for further evaluation. Moreover, the cutoff value would be obtained by statistic models, such as receiver operator characteristic (ROC) analysis, the area under thecurve (AUC) at different cutoff values for survival time was calculated, which has been used in our previous studies [33], [34]. Therefore, additional studies with the larger sample sizes, high quality and different ethnic background are needed to make a more definitive conclusion.

In summary, our meta-analysis showed that SOX2 expression was not correlated with clinicopathological parameters except for histology. Simultaneously, SOX2 overexpression predicted a betteroverall survival despite of histology. Therefore, it is appropriate to regard SOX2 expression as a promising prognostic biomarker for NSCLC patients. Prospective studies relating SOX2 expression with surgery, chemotherapy and biologicals in NSCLC are warranted.
